# Milky fluid elicited by cellulose triacetate membrane dialyzer, hyperlipidemia, and elevated C-reactive protein

**DOI:** 10.1080/0886022X.2020.1744450

**Published:** 2020-04-21

**Authors:** Shingo Watanabe, Masanori Morita, Noriyuki Hirabayashi, Tadashi Yoshida

**Affiliations:** a Medical Engineering Center, Keio University Hospital, Tokyo, Japan; b Apheresis and Dialysis Center, Keio University School of Medicine, Tokyo, Japan

**Figure 1.**
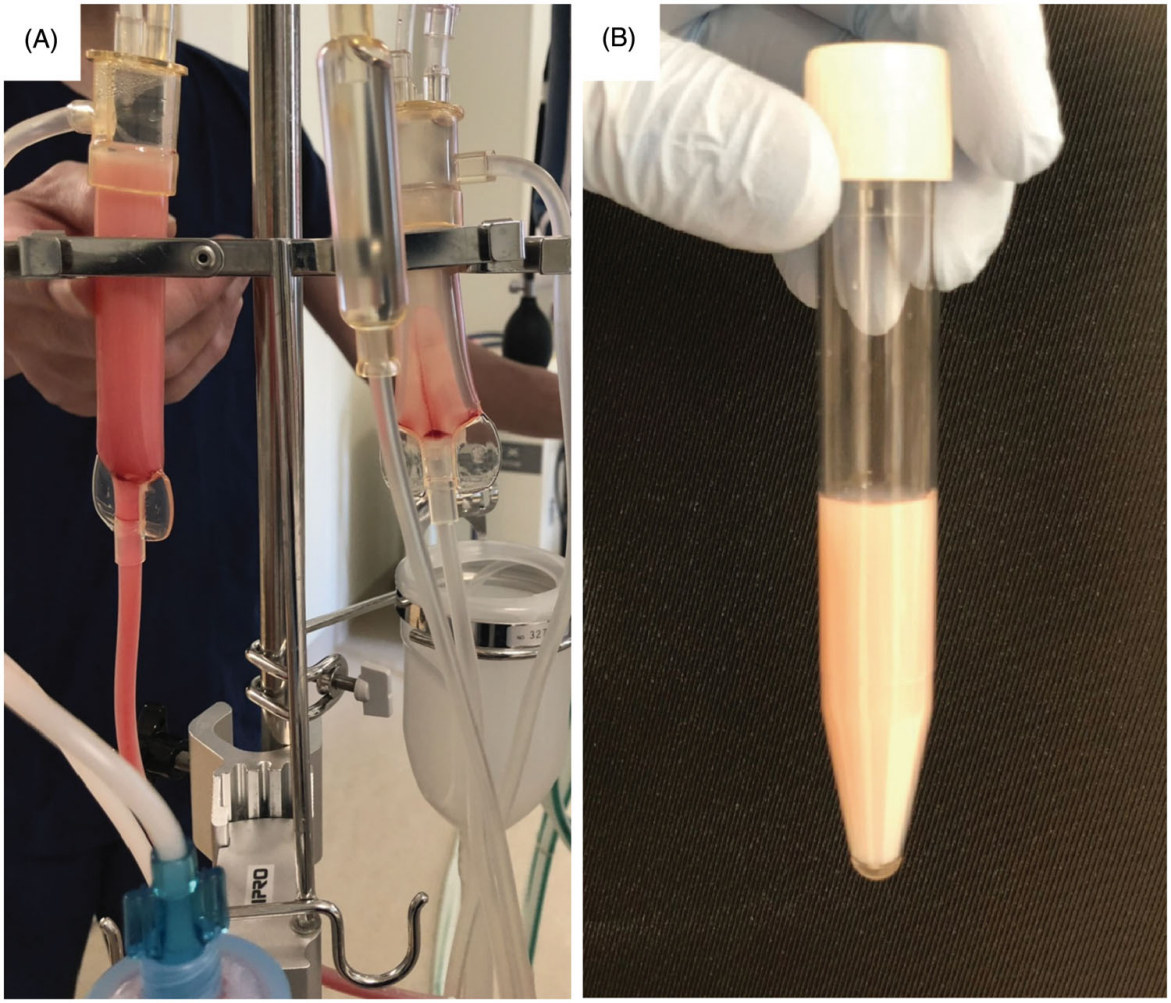
Milky fluid appeared on the venous side of the hemodialysis circuit. (A) Milky fluid was seen on the venous chamber (left) of the hemodialysis circuit, whereas it was not on the arterial chamber (right). (B) Milky fluid collected in the tube.

A 47-year-old man with malignant lymphoma and secondary membranous nephropathy was admitted to our hospital due to fever and dyspnea. He was diagnosed as having Pneumocystis pneumonia, and treatment with sulfamethoxazole and trimethoprim was begun. His renal function gradually deteriorated during the course of the treatment, and hemodialysis was thus initiated using the cellulose triacetate membrane dialyzer. Hemodialysis was stably performed at first two sessions, but pneumonia worsened. In spite of no changes in the hemodialysis settings and conditions, milky fluid appeared on the venous side of the hemodialysis circuit, when the blood was returned with 0.9% saline at the end of the third session of hemodialysis (Figure 1). The fluid in the arterial side of the circuit kept clear. We stopped returning of the blood immediately.

**Figure 2.**
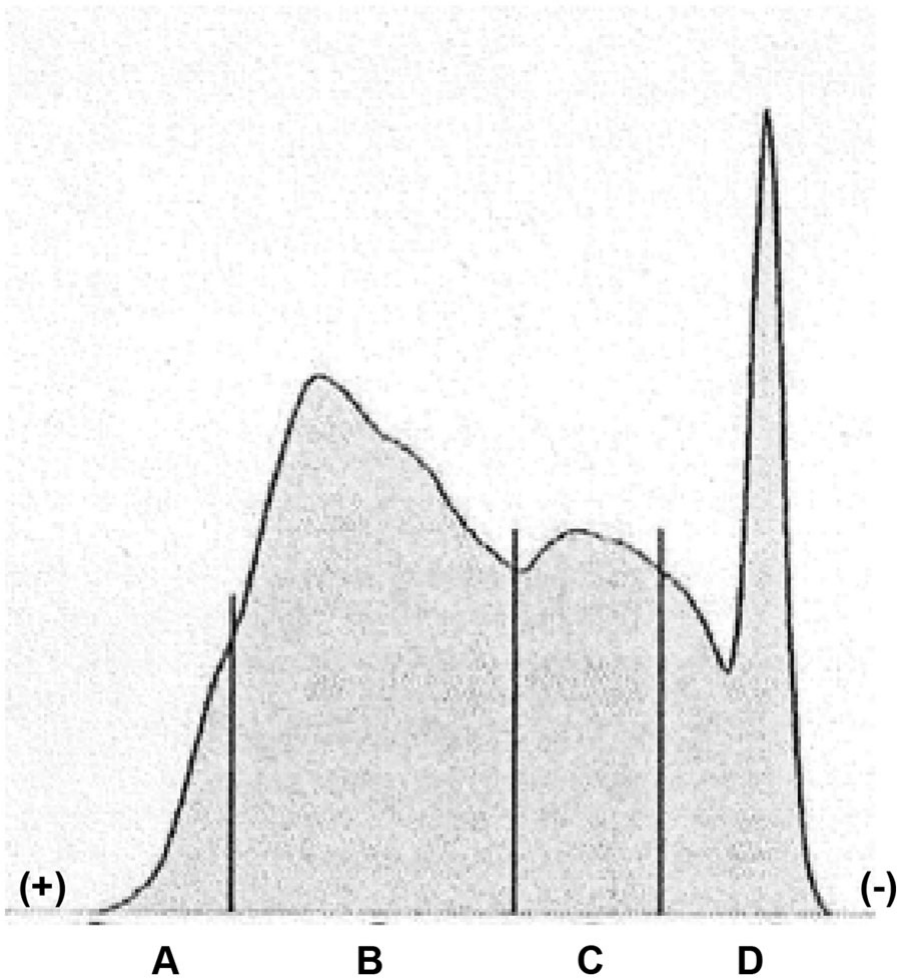
Lipoprotein electrophoresis of the milky fluid. (A) high density lipoprotein, (B) very low-density lipoprotein, (C) low density lipoprotein, (D) chylomicron.

The results of lipoprotein electrophoresis of the fluid revealed that it was rich in chylomicron and very low-density lipoprotein (Figure 2). Laboratory examination showed his serum triglyceride level of 523 mg/dL. In addition, serum level of C-reactive protein at the third session of hemodialysis was extremely elevated (28.5 mg/dL), as compared with 5.9 mg/dL at the first hemodialysis session. We considered that lipoproteins, including chylomicron and very low-density lipoprotein, were trapped, accumulated, and agglutinated on the surface of the cellulose triacetate membrane dialyzer during hemodialysis and they were released by 0.9% saline at the end of hemodialysis. In support of this, results of previous studies showed that, as compared with the polysulfone-based membrane, the cellulose triacetate membrane was able to adsorb many apolipoproteins, including apolipoprotein A-I, A-IV, C-II, C-III and E, which are the components of lipoproteins [1,2]. Elevated concentrations of C-reactive protein would also be a crucial factor enhancing agglutination of lipoproteins [3]. Based on the consideration described above, the forth session of hemodialysis was performed with the polysulfone membrane dialyzer. No milky fluid was observed, whereas his C-reactive protein level remained high. Because milky solutions may elicit the embolism in the microvessels, clinicians should be aware that the combination of the cellulose triacetate membrane dialyzer, hyperlipidemia, and elevated C-reactive protein is a potential risk for this phenomenon.

Shingo Watanabe, Masanori Morita, and Noriyuki Hirabayashi *Medical Engineering Center, Keio University Hospital, Tokyo, Japan* Tadashi Yoshida *Apheresis and Dialysis Center, Keio University School of Medicine, Tokyo, Japan* tayoshida-npr@umin.ac.jp
